# Pilocarpine-induced seizures associate with modifications of LSD1/CoREST/HDAC1/2 epigenetic complex and repressive chromatin in mice hippocampus

**DOI:** 10.1016/j.bbrep.2020.100889

**Published:** 2020-12-29

**Authors:** Verónica Noches, Carlos Rivera, Marcela P. González, Gianluca Merello, Montserrat Olivares-Costa, María Estela Andrés

**Affiliations:** Department of Cellular and Molecular Biology, Faculty of Biological Sciences, Pontificia Universidad Católica de Chile, Santiago, Chile

**Keywords:** Pilocarpine, Status epilepticus, LCH complex, H3K4me2, H3K9me2, Muscarinic receptors, Pilo, Pilocarpine, SMN, Scopolamine Methyl Nitrate, LSD1, lysine-specific demethylase 1, CoREST, Corepressor for element-1 silencing transcription factor, HDAC, Histone deacetylase, H3K4me2, histone H3 lysine 4 dimethylation, H3K9me2, histone H3 lysine 9 dimethylation, H3K4me3, histone H3 lysine 4 trimethylation, H3K9me3, histone H3 lysine 9 trimethylation, HP1α, heterochromatin protein 1α, H3ac, Histone H3 acetylated, LCH Complex, LSD1/CoREST/HDACs complex, TLE, Temporal Lobe Epilepsy

## Abstract

Epilepsy is a neurological disorder of genetic or environmental origin characterized by recurrent spontaneous seizures. A rodent model of temporal lobe epilepsy is induced by a single administration of pilocarpine, a non-selective cholinergic muscarinic receptor agonist. The molecular changes associated with pilocarpine-induced seizures are still poorly described. Epigenetic multiprotein complexes that regulate gene expression by changing the structure of chromatin impose transcriptional memories. Among the epigenetic enzymes relevant to the epileptogenic process is lysine-specific demethylase 1 (LSD1, KDM1A), which regulates the expression of genes that control neuronal excitability. LSD1 forms complexes with the CoREST family of transcriptional corepressors, which are molecular bridges that bring HDAC1/2 and LSD1 enzymes to deacetylate and demethylate the tail of nucleosomal histone H3. To test the hypothesis that LSD1-complexes are involved in initial modifications associated with pilocarpine-induced epilepsy, we studied the expression of main components of LSD1-complexes and the associated epigenetic marks on isolated neurons and the hippocampus of pilocarpine-treated mice. Using a single injection of 300 mg/kg of pilocarpine and after 24 h, we found that protein levels of LSD1, CoREST2, and HDAC1/2 increased, while CoREST1 decreased in the hippocampus. In addition, we observed increased histone H3 lysine 9 di- and trimethylation (H3K9me2/3) and decreased histone H3 lysine 4 di and trimethylation (H3K4me2/3). Similar findings were observed in cultured hippocampal neurons and HT-22 hippocampal cell line treated with pilocarpine. In conclusion, our data show that muscarinic receptor activation by pilocarpine induces a global repressive state of chromatin and prevalence of LSD1-CoREST2 epigenetic complexes, modifications that could underlie the pathophysiological processes leading to epilepsy.

## Highlights

• Pilocarpine modifies LSD1-CoREST-HDAC1/2 complexes in HT-22 cell and hippocampal neurons.
• Activation of cholinergic muscarinic receptors induces a global repressive state of chromatin in hippocampus.
• Pilocarpine-induced seizures associate to LSD1-CoREST-HDAC1/2 modifications and repressive chromatin.

## Graphical abstract

**Figure:**
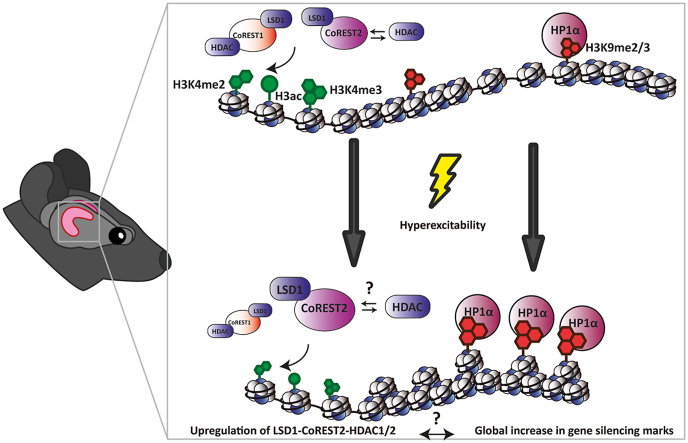

## 1 Introduction

Epilepsy is a brain disorder characterized by excessive neuronal activity and synchronized firings of some neuronal populations of the Central Nervous System that generate epileptic seizures. One central feature of epilepsy is the recurrence of epileptic seizures and the greater probability of having a new seizure after having suffered one [1,2]. Temporal lobe epilepsy (TLE) is the most common form of epilepsy and involves neuronal hyperexcitability in temporal lobe regions [3,4]. One of the animal models that best recapitulate the characteristics of TLE is the acute injection of pilocarpine in mice and rats. Pilocarpine is a muscarinic acetylcholine receptor (mAChRs) agonist, which can cause an asymmetry in the frequency of excitatory and inhibitory postsynaptic currents [5]. This initial effect can rapidly produce a *status epilepticus,* which can then develop recurrent spontaneous seizures, constituting a valuable model to study acute and chronic phases of epilepsy [6]. Although significant progress has been made in discovering neurochemical and structural changes using this model of epilepsy, many questions remain about the molecular mechanism of epileptogenesis and which epigenetic complexes are involved in initial changes that may underlie a chronic phase of the disease.

Several lines of evidence focused on revealing the molecular mechanisms of TLE have shown that histone modifications are implicated in the pathophysiology of epilepsy, highlighting the eventual role of proteins that regulate gene expression such as Methyl-CpG Binding Protein 2 (MeCP2), Repressor Element-1 Silencing Transcription Factor (REST) and others [7]. In this regard, the lysine-specific demethylase 1 (LSD1/KDM1A) has emerged as a pivotal enzyme for its role in modulating the expression of neuronal genes related to excitability and plasticity.

LSD1 is a ubiquitous epigenetic enzyme that demethylates mono and dimethylated lysines 4 and 9 in the histone H3 favoring transcriptional repression or activation, respectively [8,9]. LSD1 acts in concert with histone deacetylases HDAC1/2 as a transcriptional co-repressor complex when bound to the CoREST family of chromatin connecting proteins, constituting the LSD1-CoREST-HDAC1/2 (LCH) complex. This complex has an essential role restricting the transcription of neuro-specific genes in non-neuronal cells and neuronal stem cells [8,10] but, interestingly, all of its components are still expressed in the adult brain [11], suggesting there could be additional functions in mature cells of the central nervous system. The CoREST family of transcriptional corepressors also comprises CoREST2 (RCOR2) and CoREST 3 (RCOR3) [12,13]. Previously, we described that LCH complexes formed by the different CoREST display differential biochemical properties. In this regard, CoREST2 has less repression capacity and establishes a weaker interaction with HDAC1/2 [14], suggesting that CoREST2-containing complexes might play different roles than CoREST1-containing complexes [11]. Interestingly, it has been shown that HDAC2 protein levels increase in patients suffering from TLE [15]. Furthermore, it has been reported that LSD1 is subjected to tissue-specific alternative splicing in neurons. The expression of its neuronal specific variant (nLSD1) is transiently downregulated in the hippocampus of mice having pilocarpine-induced seizures. Moreover, mice null for nLSD1 have lower sensitivity to pilocarpine-induced seizures [16].

Considering the role of the LCH complex in regulating the expression of neuronal excitability genes, we hypothesized that changes in the expression of its components could account for the epigenetic events associated with the chromatin status in the early phase of epileptogenesis. We aimed to expand the knowledge related to the epigenetic nature of epileptogenesis by focusing on the description of chromatin changes occurring after an epileptic seizure and the enzymes involved in this process. To this end, we studied the expression of LCH components and epigenetic marks in the hippocampus of mice scoring between 2 and 5 on the Racine scale [17] after a single injection of pilocarpine. Besides, we studied the effect of pilocarpine in isolated neurons and the HT-22 hippocampal cell line, allowing determining direct muscarinic receptors activation effects. The main finding of our work indicates that the activation of muscarinic receptors leads to a repressive state of chromatin characterized by a decrease in active epigenetic marks and an increase in repressive ones, together with changes in the protein levels of the main components of the LCH complex.

## 2 Materials and methods

### 2.1 Animals

Fifty-six male C57BL/6 mice, six weeks old (21–25 g) and three pregnant adult Sprague–Dawley rats (250–300 g) were obtained from the animal facility of the Faculty of Biological Sciences of the Pontificia Universidad Católica de Chile.

All experimental procedures (protocol ID 03082015) were approved by the Bioethical Committee of the Faculty of Biological Sciences of the Pontificia Universidad Católica de Chile. All procedures were conducted to reduce the number of mice used when possible and to reduce their pain level and discomfort as much as possible. Animals were grouped in a climate-controlled vivarium on a 12 h light/dark cycle with food and water provided *ad libitum*.

### 2.2 Pilocarpine model

Animals were injected with the muscarinic receptor antagonist scopolamine methyl nitrate (SMN, Sigma–Aldrich; 1 mg/kg; i. p.) to minimize peripheral pilocarpine-induced side-effects. Pilocarpine (Sigma–Aldrich; 300 mg/kg; i. p.) was injected 30 min after SMN. We selected this dose of pilocarpine because it induced seizures in all mice and did not require the administration of anticonvulsants. The mortality rate was 9.09%. Control animals were injected with SMN, and 30 min later, an equivalent volume of the pilocarpine solution vehicle (0.9% NaCl) was injected. All animals were videotaped for 2 h to register their behavior and the severity of seizure was evaluated using the Racine scale [17].

### 2.3 Hippocampal extracellular recording

These experiments were carried out in three mice anesthetized with urethane (1.5 g/kg, intraperitoneal, ethyl carbamate, Sigma), using the protocol of Meza et al., 2018 [18]. The electrical activity was recorded by inserting a 1–1.5-μm diameter, 0.5 MΩ glass electrode in the hippocampus following the coordinates: AP: −2.0 mm; ML: 1.0 mm and DV: 2.0 mm from bregma, according to the atlas of Franklin and Paxinos [19].

### 2.4 Cell culture and primary culture of hippocampal neurons

HT-22 mouse hippocampal neuronal cell line (SCC129 Sigma-Aldrich) were cultured in Dulbecco's modified Eagle's high-glucose medium (DMEM, Gibco) supplemented with 2 mM of glutamine (Gibco), 10% fetal bovine serum (FBS, Gibco), 1% penicillin/streptomycin and maintained at 37 °C and 5% CO2.

Rat embryos of E18 pregnancy day were used for primary culture studies. Pregnant rats were decapitated, and embryos were dissected in Hank's medium. Embryos were decapitated with scissors and the hippocampus dissected out for cell culture, essentially as we have described [20]. Primary hippocampal neurons were plated on poly-l-lysine-coated covers and maintained in Neurobasal medium supplemented with B27, 1% penicillin/streptomycin, and 2 mM of glutamine. On the second day of culture, 2 μM Cytosine-Arabinoside (AraC) was added 24 h to inhibit glial proliferation. The cells were maintained at 37 °C and 5% CO2 during 7 days in vitro (DIV) before starting the experiments to allow the expression of the LCH complex components [11]. At DIV 7, neurons were treated with 200 μM of pilocarpine or 200 μM of pilocarpine (Sigma-Aldrich) plus 10 μM of scopolamine methyl nitrate (SMN, Sigma-Aldrich) or vehicle (0.9% NaCl) for 24 h, and then cells were fixed for immunofluorescence assays.

### 2.5 Tissue immunofluorescence

Twenty-four hours after pilocarpine injection, mice were deeply anesthetized with chloral hydrate (400 mg/kg, i. p.), and brain tissue was fixed by transcardial perfusion of physiological saline followed by 4% paraformaldehyde in phosphate buffer saline (PBS 1X), pH 7.5. Later, brains were removed and postfixed in 4% paraformaldehyde for 2 h and maintained in 20% sucrose in PBS 1X for 48 h. Brains were sectioned in 40 μm coronal slices using a cryostat (Leica CM 1510; Germany) from Bregma −1.7 mm to −2.46 mm, according to the atlas of Franklin and Paxinos [19]**.**

For immunodetection, the slices were permeabilized in 0.3% of Triton x-100 in PBS1x for 2 h and incubated in a blocking solution (0.05% of TRITON x-100/10% normal donkey serum (NDS) in PBS1x) for 2 h. The primary antibody solution was 0.05% of Triton x-100/5% NDS in PBS1x overnight at 4 °C. After washing with 0.3% of Triton x-100 in PBS1x, the slices were incubated for 2 h with the secondary antibodies in 0,05% of Triton x-100/1% NDS in PBS1x at RT in the dark. Finally, the slices were stained with 1 μg/mL Hoechst 33,342 (Abcam) and mounted on glass slides with 0.1% gelatin. After drying were mounted with Dako Fluorescence Mounting Medium (Agilent Technologies) [21].

### 2.6 Cell immunofluorescence

Cells were fixed with 4% paraformaldehyde in PBS 1X for 15 min and permeabilized with 0.25% Triton-X100 in PBS 1X for 5 min. Then, the cells were incubated with 3% BSA in PBS 1X for 1 h, followed by primary and secondary antibodies in the same solution for 1 h each, at RT in a humid chamber. Finally, the cells were stained with 1 μg/mL Hoechst 33,342 (Abcam). Coverslips were mounted on DAKO Fluorescence Mounting Medium (Agilent Technologies).

### 2.7 Image analysis

Images of 2560 × 1920 pixels from tissue and cells were acquired with an Olympus DS-Fi2 epifluorescence microscope equipped with a Nikon DS-Fi2 camera with standard QC capture software (Q-Imaging). The tissue images were acquired with appropriate 10× objectives and cells were acquired with appropriate 40× and 100X (using oil immersion). The time of exposition for each used laser was the same for vehicle and pilocarpine samples. Immunofluorescence quantification was performed using ImageJ software (National Institutes of Health, USA). Fluorescence intensity was calculated as the Mean Gray Value/Area (IntDen/Area) corrected by the background. The area selected to calculate the fluorescence intensity on dentate gyrus was from 998,520 to 1,151,360 pixels, using the same area for vehicle and pilocarpine for each antibody evaluated. Three to nine hippocampal slices were analyzed per animal per experiment. Six to eight fields of cultured neurons were analyzed by experiment, and for HT-22 cells, between thirty to hundred cells were quantified.

### 2.8 Western blotting

Extracted proteins were fractionated by 10% sodium dodecyl sulfate polyacrylamide gel electrophoresis (SDS–PAGE). Proteins were transferred to PVDF membrane (Millipore) blocked with 5% dry fat milk in TBS, 0.1% Tween 20 (TBS-T). Primary antibodies were incubated in 3% BSA in TBS-T overnight at 4 °C, and secondary antibodies were incubated in TBS-T for 45 min at room temperature and were revealed with SuperSignal West Pico Chemiluminescent Substrate (ThermoFisher Scientific, USA).

### 2.9 Acid extraction of hippocampal histones

Dissected hippocampal regions from control and pilocarpine-treated animals were trypsinized 15 min at 37 °C in PBS1X supplemented with protease inhibitors. Tissues were then mechanically homogenized by 30x syringe pressing movements. Cells were centrifuged at 1400×*g* 3 min and then nuclei were purified by hypotonic lysis as previously described [22]. Nuclei were resuspended in 0.5 N HCl – 10 V/V Glycerol and incubated at 4 °C for 90 min. Suspensions were centrifuged 10 min at 12,000×*g* and then clarified supernatants containing solubilized histones were transferred to a new tube. Histones were precipitated in 25% trichloroacetic acid, centrifuged 10 min at 12,000×*g*, and extensively washed with acetone. Finally, histones were resuspended in RIPA buffer, quantified, and then subjected to Western blot analyses.

### 2.10 Ultracentrifugation of nuclear proteins in sucrose gradients

Purified nuclei from mice hippocampus were subjected to extraction in a buffer containing 420 mM KCl. Salt-extracted material was clarified by 30 min centrifugation at 15,000×*g* and then loaded into a 5–20% Sucrose gradient prepared in a buffer containing 50 mM HEPES-KOH (pH 7.5), 1 mM (CH3COO)2 Mg, 80 mM CH3COOK and 0.5% (v/v) Triton X-100. Ultracentrifugation was performed for 16 h at 300,000×*g* at 4 °C. Fractions enriched in LSD1 were pooled and subjected to immunoprecipitation.

### 2.11 Immunoprecipitation

Nuclear extracts or LSD1-enriched fractions from sucrose gradient experiments were dialyzed against immunoprecipitation buffer (20 mM Tris-HCl pH 7.5, 150 mM NaCl, 1 mM EDTA, 1 mM EGTA, 1% NP40, 1 mM PMSF, 1 g/mL leupeptin and 1 g/mL aprotinin). Immunoprecipitation was performed using 45 μL Agarose-conjugated Protein A (Pierce #20333) and 1 g of anti-LSD1 antibody (ab17721) every 700 g protein in the input. Immunocomplexes were separated by centrifugation after 12 h of incubation at 4 °C. Beads were extensively washed against the CoIP buffer, and immunocomplexes were eluted by boiling the beads in 1X Laemmli Sample Buffer before Western blot analysis.

### 2.12 Antibodies

Mouse anti-CoREST1 (NeuroMab, 75–039); rabbit anti-CoREST2 (Sigma, HPA021638); rabbit anti-LSD1/KDM1 (Abcam, ab17721); rabbit anti-HDAC1 (Abcam, ab7028); mouse anti-HDAC2 (Abcam, ab51832); rabbit anti-H3K4me2 (Abcam, ab7766); rabbit anti-H3K9me2 (Abcam, ab1220); H3K4me3 (Abcam, ab8580); rabbit anti-H3K9me3 (Abcam, ab8898); rabbit anti-H3Ac (Upstate, Millipore 06–599); goat anti HP1α (Abcam, ab77256); rabbit anti-H3 (Abcam ab1791); mouse anti-GAPDH (Millipore, CB1001); donkey anti-rabbit AlexaFluor 488, donkey anti-mouse AlexaFluor 594, donkey anti-goat AlexaFluor 594 (Invitrogen Life Technologies, Carlsbad, CA, USA); Peroxidase-conjugated secondary antibodies (Jackson ImmunoResearch Laboratories, West Grove, PA, USA).

### 2.13 Q-PCR

Total RNA was isolated from mice hippocampus 7- or 24- hours after pilocarpine injection using Trizol reagent (Invitrogen Life Technologies) and reverse transcribed using MMulV (Thermo Scientific). Primers were based on the coding frame of the mouse rcor1 gene: F: 5′TCAGCAGACCACATCGTCAC3'; R:5′CATGAGGCTACAGTGCCCAA3'; rcor2 gene:F:5′AGGACAGAAGGGACTAGGGC3′; R:5′GTCACAGCCAGGAAGCAAGA3′; rcor3 gene: F:5′CACGGGGATGTTGGGATGAG3′ R:5′CGTGAAGTTAGGGAGGTCCG3'. Primers were manufactured by IDT™. All PCRs were performed with 5 x HOT FIREPol® EvaGreen® qPCR Mix Plus (Solis BioDyne) using a LightCycler 2 (Roche).

### 2.14 Exon inclusion frequency by relative quantity Fluorescent-PCR analysis (Rqf-PCR)

Total RNA was isolated from mice hippocampus 7 h after pilocarpine injection using Trizol reagent (Invitrogen Life Technologies), and reverse transcribed using MMulV (Thermo Scientific). Rqf-PCR was performed as previously described [23]. PCR primers were designed to amplify 8a exon region: Ex8_FW: 6-Fam-5′TCCCATGGCTGTCGTCAGCA3’; Ex11_RV:5′CTACCATTTCATCTTTTTCTTTTGG3′. The ratio of neuroLSD1/LSD1 was analyzed by peak scanner software v.1.0.

### 2.15 Statistical analysis

Non-parametric Mann-Whitney *U* test or unpaired Student's t-test, and One-way ANOVA, multiple comparisons, or Two-way ANOVA followed by Bonferroni post hoc test were used to determine the statistical significance of the differences, using Prism 7.0 software. The data they were expressed as mean ± SEM, and statistical significance was established at P < 0.05. The number of independent experiments and p values can be found in figure legends.

## 3 Results

### 3.1 Pilocarpine-induced seizures modify LCH components in mice hippocampus

**Fig. 1:**
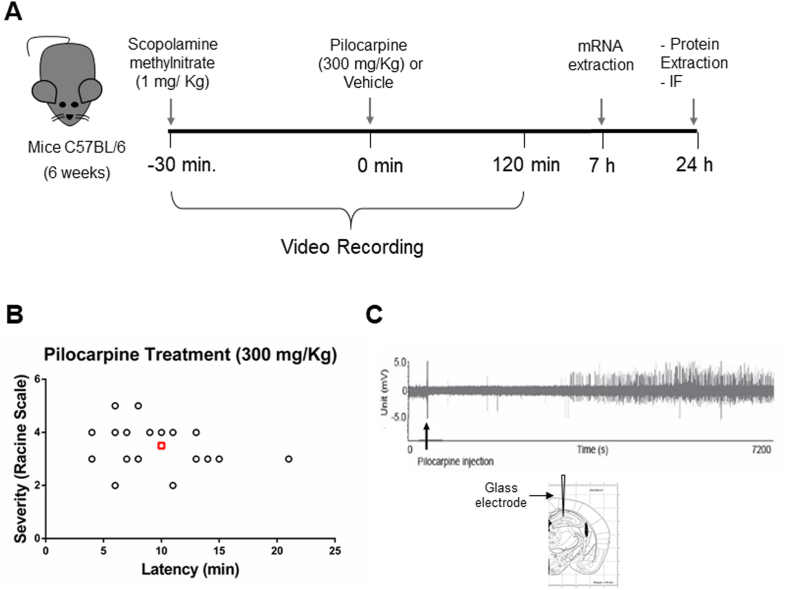
**Pilocarpine-induced seizure model.** (A) Scheme of the protocol used to treat mice with pilocarpine (300 mg/kg) and time of sample collection. Control animals were injected with vehicle, and both groups were injected 30 min before with scopolamine (SMN). Samples for mRNA analyses were taken at 7 and 24 h post pilocarpine. Proteins analysis was carried 24-h post-treatment. (B) The severity of seizure (Racine scale) and latency (min) was quantified in 22 animals treated with pilocarpine. The red square indicates the mean latency and seizure severity of the sample. (C) Extracellular recording in the hippocampus of a mouse injected with pilocarpine under anesthesia (representative example of 3 mice). Nine hundred seconds after the injection of pilocarpine, an increase in neuronal activity was observed. The position of the glass electrode in the hippocampus is shown below. (For interpretation of the references to colour in this figure legend, the reader is referred to the Web version of this article.)

The LCH complex regulates the expression of genes involved in neuronal excitability. Therefore, we wondered whether the LCH complex's components and chromatin's functional status in the hippocampus are modified in the model of epileptogenesis induced by pilocarpine. For this purpose, we first characterized the pilocarpine-induced epilepsy model in our laboratory. The protocol used consisted of an injection of pilocarpine (300 mg/kg) 30 min after injecting scopolamine (SMN) to minimize peripheral pilocarpine-induced side effects (Fig. 1A). Latency to the beginning of seizure and severity was quantified in all mice used for posterior biochemical analysis. As shown in Fig. 1B, all mice injected with this dose of pilocarpine suffered seizure. An average of 10 min of latency to first signs of a seizure and an index of 3.5 in behavioral Racine's scale was determined for the group (Fig. 1B). We corroborated that the dose of pilocarpine occupied in our model generated an increased neuronal activity by performing an extracellular recording in the hippocampus of anesthetized mice (Fig. 1C).

**Fig. 2:**
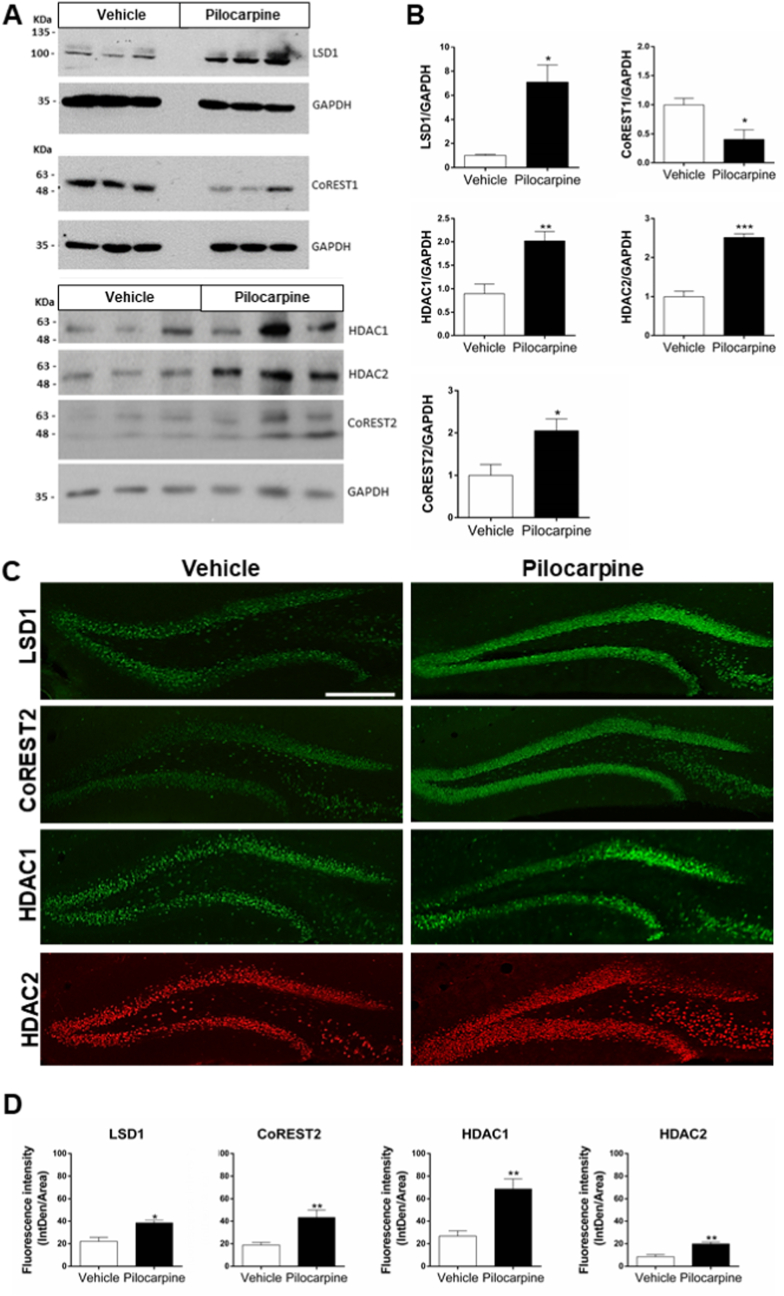
**LCH complex is modified under pilocarpine-induced neuronal hyperexcitability.** (A) Western blot analysis of LSD1, CoREST1, HDAC 1/2, and CoREST2 in total protein extracts from the hippocampus of mice 24 h after pilocarpine or vehicle injection. (B) Densitometry quantification is expressed as the ratio over GAPDH. Bars correspond to the mean ± SEM of three independent experiments. Statistical analysis performed with Unpaired *t*-test. *, P˂ 0.05; **, P˂ 0.005; ***, P = 0.0008. (C) Immunofluorescence of dentate gyrus from the hippocampus 24 h after pilocarpine or vehicle injection. Scale bar: 20 μm. (D) Quantification of immunofluorescence from LCH complex components in the dentate gyrus. Bars correspond to the mean ± SEM of three to four independent experiments. Statistical analysis performed with Mann-Whitney test. *, P ˂ 0.05; **, P ˂ 0.005.

Twenty-four hours after pilocarpine injection, the hippocampi were removed for Western blot analysis (Fig. 2A). We observed that total protein levels of LSD1, HDAC1, and HDAC2 increased after pilocarpine treatment (Fig. 2B). Interestingly, while CoREST1 decreased, CoREST2 protein levels increased with pilocarpine treatment (Fig. 2B), suggesting differential consequences in the components of LCH complexes under hyperexcitability. To determine where these modifications were occurring, mouse brain immunofluorescence analyses were performed 24 h after pilocarpine injection. Immunofluorescence was carried out in the ventral hippocampus (Fig. 2C), given the importance of this nucleus in the development of the epileptic state in this model [24]. Consistent with Western blot data, we observed an increase in LSD1, CoREST2, HDAC1, and HDAC2 in the dentate gyrus of the hippocampus (Fig. 2D). Pilocarpine-induced changes of LCH components were specific to the hippocampus, as analyzes of other brain nuclei, such as the striatum and prefrontal cortex, showed no modifications of the LCH complex components (data not shown).

**Fig. 3:**
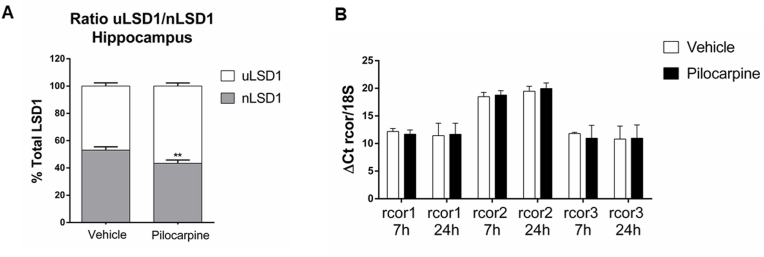
**Pilocarpine does not modify the transcripts of the rcor genes** (A) The proportion of neuronal variant (nLSD1) and ubiquitous LSD1 (uLSD1) transcripts assessed by Rqf-PCR after 7 h of pilocarpine or vehicle injection. Bars represent the mean ± SEM of six independent experiments. Statistical analysis was performed with Two-way ANOVA.**, P ˂ 0.005. (B) Q-PCR for *rcor1, rcor2,* and *rcor3* mRNAs in the hippocampus of mice after seven and 24 h of pilocarpine or vehicle injection. The 18S RNA was used as the reference transcript. Bars correspond to the mean ± SEM of three to four independent experiments.

To investigate whether the observed changes in protein levels are due to a change in transcript levels, we performed a qPCR study at short (7 h) and long times (20 h) after pilocarpine injection. Previously, it was shown that pilocarpine-induced seizures modify transiently the expression of the LSD1 neuronal variant, nLSD1 in the hippocampus [16]. We also observed the transient decrease of nLSD1 transcripts in the hippocampus 7 h after pilocarpine injection (Fig. 3A). Under the same conditions, the mRNA expression of rcor1, rcor2, and rcor3 genes remained similar to controls in the hippocampus at seven and 24 h after pilocarpine (Fig. 3B). Thus, pilocarpine-induced modifications in CoREST1 and CoREST2 protein levels were not due to transcriptional changes.

### 3.2 The interaction between CoREST1 and LSD1 is maintained after pilocarpine treatment

**Fig. 4:**
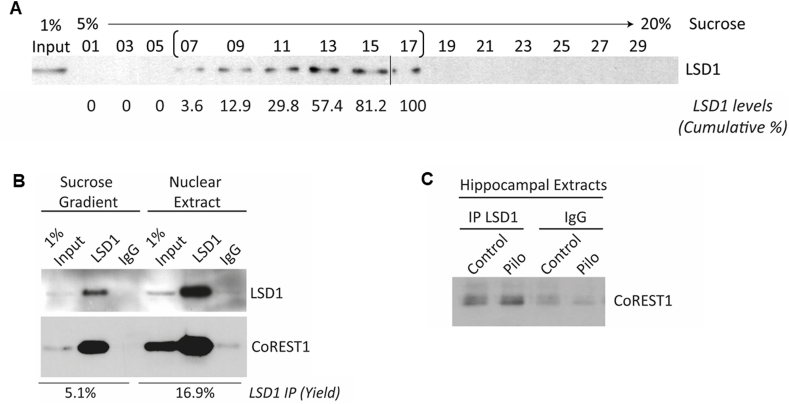
**Immunocomplexes formed between CoREST1 and LSD1 in the hippocampus are maintained after pilocarpine treatment.** (A) Western blot analysis of LSD1 in a sucrose gradient from purified nuclei from three mice hippocampus. Fractions [07 to 17] which concentrated 100% of detected LSD1 (as shown under fraction analyses as cumulative LSD1 content), were pooled and subjected to immunoprecipitation. (B) Western blot analysis of co-immunoprecipitation from fractions enriched in LSD1 after sucrose gradient or directly from nuclear extracts. LSD1 immunoprecipitation yields are shown under the blots and expressed as the percentage of total LSD1 enriched after IP. IgG: Immunoglobulin G used like specificity control. (C) Western blot analysis of co-immunoprecipitation of LSD1 and CoREST1 in hippocampal chromatin from mice 24 h after pilocarpine or vehicle injection.

To assess the integrity of the LCH complex against pilocarpine-induced seizure, we tested whether immunocomplexes formed between endogenous CoREST1 and LSD1 proteins in the hippocampus were modified after pilocarpine injection. To this end, we biochemically enriched hippocampal LSD1 after separating hippocampal nuclear proteins on a 5–20% sucrose gradient and pooling LSD1-enriched fractions (Fig. 4A). Next, immunoprecipitation experiments were performed either on soluble nuclear extracts or LSD1-enriched sucrose gradient fractions (Fig. 4B). As previously shown [[12], [13], [14]], we observed that LSD1 interacts with endogenous CoREST1 (Fig. 4B), indicating that the complex LSD1-CoREST1 is present in the hippocampus adult mouse. Twenty-four hours after pilocarpine injection, the interaction between LSD1 and CoREST1 was maintained similar to controls in hippocampal chromatin (Fig. 4C), indicating that pilocarpine treatment does not interrupt the interaction between LSD1 and CoREST1.

### 3.3 Epigenetic changes occurring after pilocarpine treatment

**Fig. 5:**
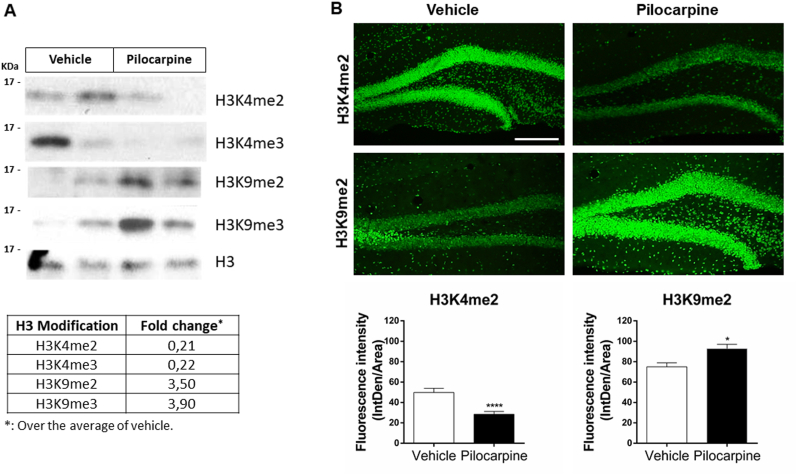
**Pilocarpine treatment affects the global state of chromatin.** (A) Western blot of acid-extracted hippocampal histones and analysis of active (H3K4me2/3) and repressive (H3K9me2/3) H3 modifications from pilocarpine and vehicle-injected mice. H3 is used as loading control. Fold change was calculated by densitometry and normalized concerning the average of the two controls. (B) Immunofluorescence of H3K4me2 and H3K9me2 in the dentate gyrus from the hippocampus of mice 24 h after pilocarpine or vehicle injection. Bars represent the mean ± SEM of three independent experiments. Scale bar: 20 μm. Statistical analysis performed by Mann-Whitney test. *, P ˂ 0.05; ****, P < 0.0001.

Changes in the protein levels of LCH-complex components prompted us to study whether the histone substrates of LCH complexes are also affected by the pilocarpine treatment. The evidence accumulated so far shows that LSD1 can demethylate H3K4me1/me2 and H3K9me1/me2, inducing gene repression and activation, respectively [8,9,25]. Hence, we carried out Western blot analyses of acid-extracted hippocampal histones and detected a tendency of pilocarpine to decrease the levels of active H3 modifications (H3K4me2/3) and to increase the repressive H3 modifications (H3K9me2/3) (Fig. 5A), suggesting that pilocarpine induces a global change in chromatin architecture. We then confirmed by immunostaining an increase in the epigenetic mark of inactive chromatin (H3K9me2) and a decrease in the epigenetic mark of active chromatin (H3K4me2) in the hippocampus of mice treated with pilocarpine, 24 h after the injection (Fig. 5B). Altogether, these data indicate that pilocarpine induces changes in components of LCH complexes and increased epigenetic marks of repressed chromatin.

### 3.4 Cholinergic control of the transcriptional repressor complex LCH

**Fig. 6:**
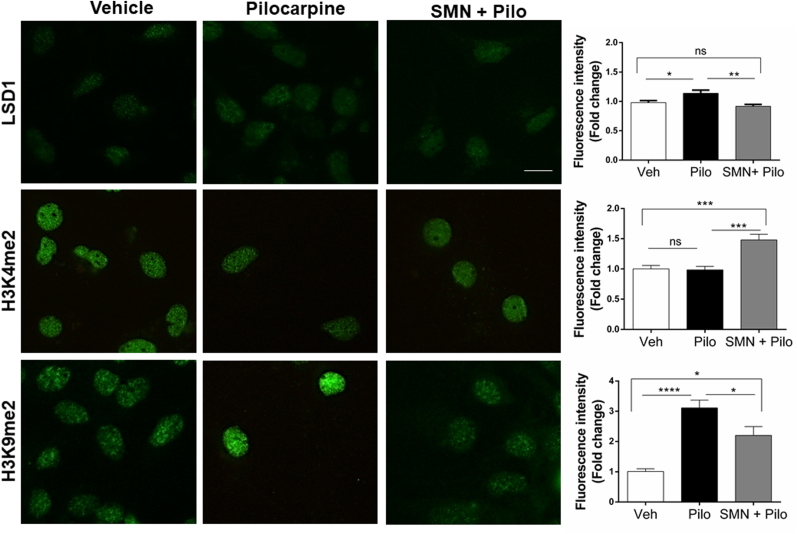
**Muscarinic receptors mediate the modifications of LSD1 and epigenetic marks induced by pilocarpine.** Immunofluorescence of LSD1, H3K4me2, and H3K9me2 in DIV7 of hippocampal primary cultures treated with pilocarpine (200 μM) and/or scopolamine methyl nitrate (SMN, 10 μM) for 24 h. Scale bar: 5 μm. Fold change expressed over the vehicle. Bars represent the mean ± SEM of three independent experiments. Six to eight fields were quantified in each condition. Statistical analysis performed with One-way ANOVA, multiple comparisons. *, P ˂ 0.05; ***, P = 0.0006; ****, P < 0.0001.

To test the idea that the LCH complex is a target of the cholinergic system, we evaluated the effect of pilocarpine and SMN, a specific antagonist of M1/M2 receptors, in isolated hippocampal neurons. To achieve this goal, rat primary cultures at DIV7 of E18 embryos were treated during 24 h with pilocarpine (200 μM) alone or in the presence of SMN (10 μM), and then subjected to immunofluorescent analysis. Consistent with in vivo data, pilocarpine increased LSD1 protein levels in cultured hippocampal neurons, effect that was reversed by SMN. Similarly, increased H3K9me2 induced by pilocarpine was reversed by SMN (Fig. 6). On the other hand, the epigenetic mark of active chromatin (H3K4me2) did not change with pilocarpine but increased in the presence of SMN. Together, these data suggest that the cholinergic system through muscarinic M1/M2 receptors regulates LSD1 levels and chromatin status, promoting a repressive transcriptional state.

**Fig. 7:**
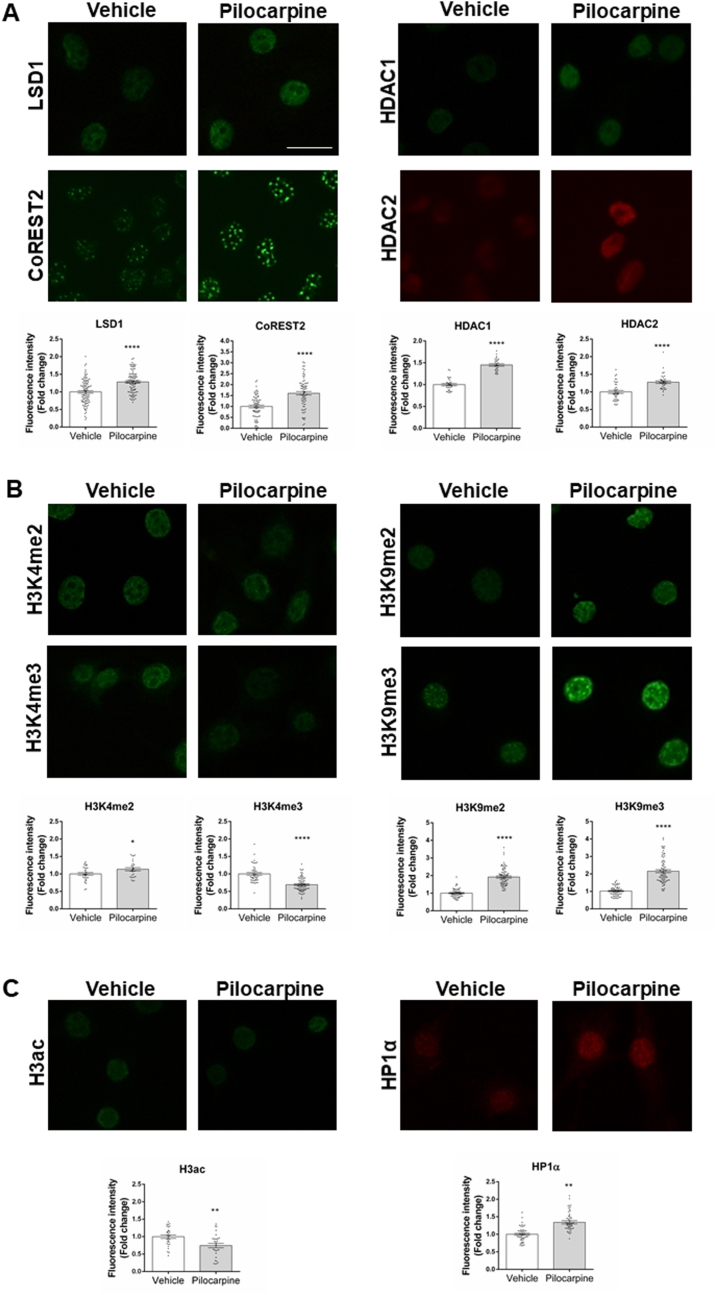
**Pilocarpine induces modification of LCH components in HT-22 cells.** (A) Immunofluorescence of LSD1, CoREST2, and HDAC1/2. (B) Immunofluorescence of epigenetic marks, H3K4me2/me3, H3K9me2/me3. (C) Immunofluorescence of H3 acetylated, and heterochromatin protein 1α (HP1α) 24 h after pilocarpine or vehicle treatment. Scale bar: 10 μm. Fold change expressed over the vehicle. Bars represent the mean ± SEM of thirty to one hundred cells. Statistical analysis was performed with an Unpaired *t*-test. *, P ˂ 0.05; **, P ≤ 0.003; ****, P < 0.0001.

To further inquire in the role of muscarinic receptors regulating LCH complex components and epigenetic status of chromatin, we tested the effect of pilocarpine in HT-22, a hippocampal cell line that expresses M1 and M2 muscarinic receptors [26]. Similar to the in vivo findings, treatment of HT-22 cells with pilocarpine increased the protein levels of LSD1, CoREST2, and HDACs1/2 (Fig. 7A). Likewise, pilocarpine induced a repressive state of chromatin in HT-22 cells characterized by lower levels of the epigenetic mark H3K4me3 (Fig. 7B) and reduction in acetylated histone H3 (Fig. 7C). Furthermore, we detected an increase in the heterochromatin protein 1α (HP1α) (Fig. 7C), which is the reader of the H3K9me2/3 marks [27], associated with higher levels of H3K9me2/3 in pilocarpine-treated HT-22 cells (Fig. 7B). Together these data suggest that activation of muscarinic cholinergic receptors M1 and M2 induces a state of transcriptional repression of chromatin and changes in the LCH complex.

## 4 Discussion

This study provides evidence that the cholinergic system regulates components of the LSD1-CoREST-HDAC1/2 complex through M1/M2 muscarinic receptors. We detected changes in main components of the LCH complex produced by pilocarpine in vivo, in cultured neurons, and HT-22 cells, and global changes in chromatin structure revealed by increased levels of repressive histone modifications and decreased activating marks.

LSD1, when bound to CoREST proteins, is a transcriptional corepressor that removes methyl groups from lysine residues in chromatin histones whose methylation is linked to active transcription [8]. Pilocarpine treatment in the animal caused an increase in the protein levels of LSD1 and CoREST2 and a decrease of CoREST1 in the hippocampus. This data suggests the existence of a novel post-translational mechanism, which might stabilize LSD1, CoREST2, and HDAC1/2 levels upon cholinergic activation. Interestingly, while CoREST1 depleted cells have been shown to down-regulate LSD1 [12], LSD1 levels did not decrease in the pilocarpine-induced epilepsy model, which produced CoREST1 down-regulation. Furthermore, since we could not detect changes in LSD1-CoREST1 interaction, we speculate that the excess of CoREST2 might be establishing new complexes with increased LSD1 after pilocarpine injection. Previous evidence suggested a complementary role for CoREST1 and CoREST2 because of their high sequence homology; CoREST2 is also present in complexes with LSD1 and weakly with HDAC1/2 [14], and while knockout animals for rcor1 or rcor2 are viable, the rcor1/2 double knockout animal dies between E18.5 and P1 [28]. Thus, our data suggest that CoREST proteins can be differentially modulated under hyperexcitability and CoREST2 might be stabilizing the observed LSD1-excess.

HDACs have a crucial role in suppressing gene transcription by condensing the chromatin structure, removing the acetyl groups of the amino-terminal lysines from the histone tails, which gives histones a positive net charge, making it more compact, characteristic of gene repression [29,30]. This global repression was associated with an increase of repressive marks (H3K9me2/3) and HP1α, also induced by pilocarpine. We observed that HDAC1/2 increased in the hippocampus after 24 h of a pilocarpine injection as was reported by Huang et al. [15]. We speculate that HDAC1/2 increased activity may aid in the establishment of a global repressive state by complexes that impose repressive methylations on histone H3, as it has been shown when HDACs facilitates H3K27me3 and H3K9me3 catalyzed by EZH2 and G9a, respectively [[31], [32], [33]].

A deficiency of HDAC2 is known to cause an increase in the number of synapses and the facilitation of memory [34]. After pilocarpine treatment, we detected a sharp increase in HDAC1/2 and a decrease in acetylated H3. There is evidence that HDAC inhibition prevents the development and persistence of TLE [35], and a decrease in acetylated H3 is implicated in the development of epilepsy [36].

Interestingly, genome-wide profiling studies of hippocampal gene expression changes on animals subjected to pilocarpine treatments have shown that a large subset of genes change their pattern of expression at different time points after presenting a status epilepticus, suggesting that a global and dynamic reprogramming of gene expression is occurring [37]. Similar results were obtained on rats, where chronic induction of epilepsy was reported to induce a significant increase in global DNA methylation marks, mostly enriched in CpG islands and genic regions [38]. These observations support that a global repressive environment must be imposed in the hippocampal genes. Therefore, our results support that model as we observed a global increase in repressive histone marks, occurring at the early stages of epileptogenesis.

The state of the chromatin and the components of the LCH repressor complex change as a result of the neuronal hyperexcitability produced by pilocarpine. Interestingly, these changes principally occurred at the dentate gyrus of the hippocampus, a region that has high expression levels of M1 muscarinic receptors compared to others [39]. Our in vivo and in vitro data suggest that the mechanism underlying these modifications is modulated through the activation of muscarinic receptors. Sixty days post-seizure pilocarpine-induced the HDAC2 increased expression is maintained [15]. Thus, given that LSD1, CoREST2, and HDAC1/2 increased after pilocarpine treatment, they could be participating in establishing epigenetic modifications necessary to develop a chronic epileptic phenotype. In this regard, it is worth mentioning that the LCH complex is recruited by the REST (NRSF) transcription factor to regulate the expression of a significant number of genes involved in excitability, neurogenesis, among other processes [40]. It has been reported that the expression of REST increases in the hippocampus in several models of epileptogenesis [41]. Our data showing that the LCH complex, required for REST to exert its repressive transcriptional effect on target genes, also increases, reinforces the idea of a functional REST during the early phases of epileptogenesis. Whether REST-LCH complex plays a protective or inductive role in the intermediate and chronic phases of epileptogenesis deserves further investigation.

## Funding

We gratefully acknowledge funding by the National Agency for Research and Development (ANID), Chile (FONDECYT Postdoctoral #3160308; FONDECYT Regular #1191152 and Doctoral fellowship #21161044).

## Author contribution

V·N and C.R conceived and designed the study, participated in the acquisition, analysis, interpretation of data, and the manuscript's writing. M.P.G., G.M and MOC participated in data acquisition and the editing of the manuscript. M.E.A. supervised the development of the project, contributed to the writing, reviewed, and approved the manuscript's final submission.

## Declaration of competing interest

The authors declare that they have no known competing financial interests or personal relationships that could have appeared to influence the work reported in this paper.
